# Serum amyloid a production in chicken embryonic synovial fibroblasts induced by lipopolysaccharide, interleukin-1β, and vitamin A

**DOI:** 10.1016/j.psj.2025.105358

**Published:** 2025-06-02

**Authors:** Belma Dayı, Alper Sevimli, Ahmet Akkoç, Ayşe Meriç Mutlu, Nurhan Doğan

**Affiliations:** a Department of Pathology, Faculty of Veterinary Medicine, Afyon Kocatepe University, Afyonkarahisar, Turkey; b Department of Pathology, Faculty of Veterinary Medicine, Bursa Uludag University, Bursa, Turkey; c Department of Biostatistics and Medical Informatics, Faculty of Medicine, Afyonkarahisar Health Sciences University, Afyonkarahisar, Turkey

**Keywords:** Chicken embryonic synovial fibroblast, Interleukin-1β, Serum amyloid A, Vitamin A, qPCR

## Abstract

Amyloid arthropathy, which negatively affects animal welfare by causing various health problems in brown laying hens, is a pathological phenomenon that occurs when amyloid A (AA) protein accumulates in the leg joints and serum amyloid A (SAA), the precursor of AA protein, forms permanent amyloid fibrils. This study aimed to evaluate the SAA production at the 24th and 48th h by enzyme-linked immunosorbent antibody assay (ELISA) in culture medium and real-time quantitative polymerase chain reaction (qPCR) after the induction of chicken embryonic synovial fibroblasts (CESF) with different doses of lipopolysaccharide (LPS), interleukin-1β (IL-1β) and vitamin A. Hemacolor staining, immunocytochemistry, and carbon powder uptake test were performed to characterize the isolated CESF in the study. A statistically significant increase was observed in SAA mRNA expression in all groups at the 24th and 48th h, except the group induced with vitamin A for 48 h, while no increase was detected in the SAA level by ELISA. In the qPCR results, the highest increase among the 24-hour groups was 2604.5 ± 476 in the LPS (10 μg/mL) + IL-1β (30 ng/mL) applied group, while the highest increase among the 48-hour groups was 1577.4 ± 326.4 in the LPS (5 μg/mL) + IL-1β (50 ng/mL) + Vitamin A applied group. The results have shown that SAA expression in CESF is particularly dependent on LPS concentration, with IL-1β and vitamin A being less effective CESF from the brown layer hen embryos proved to be a suitable study model for amyloid arthropathy.

## Introduction

Amyloid is a protein with an abnormal structure that cannot be dissolved by proteolytic enzymes. The accumulation of this substance in a permanent fibrillar structure among cells is called amyloidosis (Landman et al., 1998). Amyloidosis in poultry is a complex phenomenon observed in conditions such as chronic infections and inflammatory reactions, but the pathogenesis is not yet fully known (Landman et al., 1999). It is notable that all amyloidosis cases reported in poultry are secondary amyloidosis caused by amyloid A (AA) protein accumulation (Landman et al., 1996). It has been noted that the most common amyloidosis in chickens is amyloid arthropathy, which is one of the most significant leg diseases in poultry (Landman et al., 1994; Steentjes et al., 2002).

AA protein accumulates in the joints in amyloid arthropathy (Landman et al., 1994). The disease can cause growth depression, decreased egg production, and lameness in approximately 20-30 % of the chickens in a commercial flock, especially in brown laying hens (Landman et al., 1994). It is known that serum amyloid A (SAA), the precursor of AA protein, and proinflammatory cytokines such as interleukin-1β (IL-1β), interleukin-6 (IL-6), tumor necrosis factor-α (TNF-α) are the leading predisposing factors in amyloid formation (Bochsler and Slauson, 2002; Landman et al., 1998). Additionally, agents such as *Enterococcus faecalis* (Peperkamp et al., 1997; Sevimli et al., 2004), *Mycoplasma synoviae* (Liu et al., 2021), Freund's adjuvant (Sevimli et al., 2005), lipopolysaccharide (LPS) (Jacobsen et al., 2006; Koziy et al., 2024; Upragarin et al., 2005) and vitamin A (Sevimli et al., 2005) have been reported as important inducers of SAA. However, the primary roles of these agents in the formation of amyloid arthropathy are still controversial (Landman et al., 1998).

Synovial fibroblasts (SF), which play a very important role in joint diseases, are located in the intima layer of the synovial membrane. It causes an ongoing inflammatory reaction by releasing pro-inflammatory mediators as a result of the influence of noxious stimuli (Ritchlin, 2000; Yoshitomi, 2019) and plays an active role in local SAA synthesis (Blanco et al., 2016; Liu et al., 2021; Upragarin et al., 2005).

Literature reviews show that studies on amyloid arthropathy, have primarily been conducted *in vivo*. To our knowledge, only one study has investigated the release of SAA from chicken SF *in vitro* (Upragarin et al., 2005). However, there are no studies which have been conducted to examine the release of SAA from SF of chicken embryos *in vitro*. Considering the economic importance of amyloid arthropathy, further studies are needed to obtain detailed information about the pathogenesis and to reduce the economic losses caused by secondary amyloidosis in poultry (Blanco et al., 2016; Sevimli et al., 2013; Upragarin et al., 2005). One of our hypotheses in this study was that SF located in the intima layer of the synovial membrane plays an important role in joint diseases and using these cells may be an effective method to detect their role in amyloid arthropathy. Our aim for this hypothesis was to create for the first time an *in vitro* experimental model in which SAA synthesis in CESF induced by LPS, IL-1β and vitamin A can be measured with molecular techniques. Our other hypothesis was that evaluating the effect of different agents on SAA synthesis by SF in terms of dose and duration may enable the development of diagnostic and therapeutic approaches for the pathogenesis of amyloid arthropathy. Our aim for this hypothesis was to evaluate the production of SAA at the 24th and 48th h in CESF induced by different doses and agents by enzyme-linked immunosorbent antibody assay (ELISA) and real-time polymerase chain reaction (qPCR).

## Materials and methods

### Cell culture

For the study, specific pathogen-free eggs of the brown layer hen (ISA Brown) were obtained from a commercial hatchery flock (Güres Yumurta A.S., Turkey). After the eggs were transferred to the laboratory, they were incubated at 37°C in a humidified 5 % CO_2_ atmosphere (Cimuka, PD60, Turkey) for 11 days. At the end of the incubation period, the eggs cleaned with 70 % alcohol were opened, and embryos were taken into sterile petri dishes. Decapitation method was used for embryo euthanasia (IACUC, 2016). After euthanasia, 0.5 cm x 0.5 cm tissue samples were taken from the tibio-metatarsal joints of the embryos. The samples were placed in petri dishes filled with 50 mL Dulbecco’s phosphate buffered saline (DPBS) solution (Sigma Aldrich, D5662, USA) containing 2X penicillin-streptomycin (Sigma Aldrich, P4333, USA). CESF was isolated using a procedure previously described by Dayı et al. (2023). Briefly, samples taken from the tibio-metatarsal joint region were dissected in a class II biosafety cabinet (Jouan, France) and placed in 6-well cell culture plates (TPP, 92006, Switzerland). Two mL of M199 (Sigma Aldrich, M3769, USA) containing 20 % fetal bovine serum (FBS) (Sigma Aldrich, F2442, USA) and 2X penicillin-streptomycin was added to the explants and incubated at 37°C with 5 % CO_2_ (Jouan, France) for 48 h. The morphology and growth of the cells were observed regularly every other day with an inverted microscope (Olympus, CKX41, Japan).

In primary cell culture, cells were passaged seven days after seeding when they reached 80 % confluency. Each well in the 6-well plates was rinsed with preheated DPBS for five min. After DPBS was removed, 0.5 mL of preheated trypsin-EDTA solution (Biological Industries, 1109199, Israel) was added and the 6-well plates were incubated for two min at 37°C. Trypsin activity was stopped by adding 2 mL of M199 containing 2X penicillin-streptomycin and 20 % FBS into the wells. The cell suspension was transferred to a 25 cm^2^ tissue culture flask. Three mL of M199 was added to the flasks and the flasks were placed in the incubator at 37°C and 5 % CO_2_. When the cells reached 80 % confluency on day 12, a second passage was performed and the flasks were incubated under the same conditions as mentioned above. Then, the cell suspension was transferred to 75 cm^2^ tissue culture flasks and evaluated under an inverted microscope with regular periods.

### Characterisation of cells

To characterize the cells before the eighth passage, cells were added to round coverslips placed in 24-well plates and incubated in a humidified atmosphere of 5 % CO_2_ at 37°C overnight. Hemacolor staining was performed after the cells reached 80 % confluency. Cells were also stained immunohistochemically using monoclonal anti-vimentin and anti-cytokeratin antibodies (described below). Additionally, carbon powder uptake test was performed by adding 100 μl of the cell suspension into 24-well plates (described below).

### Hemacolor staining

Cells seeded on coverslips in 24-well plates were stained with hemacolor as described before (Dayı et al., 2023). Briefly, after washing the wells with PBS for five min, approximately 0.5 mL of cold methanol (−20°C) was added to the cells and cells were fixed for five min. Then, the methanol was removed, cells were washed with PBS for 2 × 5 min and stained with reagent red (Merck, 1.11956.2500, Germany) for 5 × 1 min and reagent blue (Merck, 1.11957.2500, Germany) for 5 × 1 min.

### Immunocytochemistry

Cells seeded on coverslips in 24-well plates were stained immunocytochemically using a commercial kit (Thermo Fisher Scientific, TP-015-HDJ, UltraVision ONE Detection System, HRP&DAB, USA), using a procedure by Dayı et al. (2023). The wells of the culture plates were rinsed with PBS for five min. 0.5 mL of cold (−20°C) methanol was added to the wells and the plates were incubated for five min. Then, methanol was removed and the wells were washed with PBS for twice for five min. The protein blocking solution was applied for 10 min. Later, monoclonal anti-vimentin (Dako/Agilent Technology, M0725) and anti-cytokeratin (Santa Cruz Biotechnology, Sc-57004) primary antibodies, which were used in chicken lung fibroblasts (Akkoc and Kahraman, 2012) and CESFs (Dayı et al., 2023), were added to cover the coverslips and the coverslips were incubated for one hour at room temperature. Secondary antibody was added to the wells, which were washed twice for five min with PBS, and incubated for 30 min. Then 3,3 diaminobenzidine (DAB) (2 mL DAB substrate solution + 40 μl DAB chromogen solution) was added for two min, followed by counterstaining with Harris hematoxylin (Merck, 1.07961, Germany) for one minute.

### Carbon powder uptake test

The phagocytic capacity of CESF and whether they were contaminated with macrophage cells were evaluated by carbon powder uptake test (Dayı et al., 2023; Upragarin et al., 2005). 50 μg/mL carbon powder (Sigma Aldrich, 05105, USA) suspension was added to 24-well plates the plates were incubated at 40°C for 30 min. At the end of the period, plates were washed with PBS for five min and examined under an inverted microscope.

### Experimental design

CESF isolated after the 8th passage was used in the experiments. The experiments were divided into groups depending on dose and duration. Respectively, 13 and 16 subgroups were created in 24 and 48-hour groups. LPS (Sigma Aldrich, L4516, *Escherichia coli* O127:B8, USA), IL-1β (Lifespan Biosciences, LS-G37646, USA) and vitamin A (Sigma Aldrich, R0635, USA) were administered to these groups separately and in combination. The substances and doses administered to each group are shown in Table 1. While the LPS group, known as an SAA inducer was used as a positive control, CESF without any application was determined as the negative control group. While creating the experimental groups, only one subgroup could be formed in the 24-hour combined group. The reason for this was that there was not enough space in the 96-well plate used in the ELISA analysis and a second kit could not be purchased due to the limited study budget.

**Table 1:** Inducing substances and their doses applied to the experimental groups.

Groups	Inducing substances at 24 h	Inducing substances at 48 h
Negative control	-	
LPS (Positive control)	LPS (5 µg/mL)	
	LPS (10 µg/mL)	
Vitamin A	Vitamin A (7 µg/mL)	
Cytokine	IL-1β (30 ng/mL)	
	IL-1β (50 ng/mL)	
LPS + cytokine	LPS (5 µg/mL) + IL-1β (30 ng/mL)	
	LPS (5 µg/mL) + IL-1β (50 ng/mL)	
	LPS (10 µg/mL) + IL-1β (30 ng/mL)	
	LPS (10 µg/mL) + IL-1β (50 ng/mL)	
LPS + vitamin A	LPS (5 µg/mL) + Vitamin A (7 µg/mL)	
	LPS (10 µg/mL) + Vitamin A (7 µg/mL)	
Combined	-	LPS (5 µg/mL) + IL-1β (30 ng/mL) + Vitamin A (7 µg/mL)
	-	LPS (5 µg/mL) + IL-1β (50 ng/mL) + Vitamin A (7 µg/mL)
	-	LPS (10 µg/mL) + IL-1β (30 ng/mL) + Vitamin A (7 µg/mL)
	LPS (10µg/mL) + IL-1β (50ng/mL) + Vitamin A (7µg/mL)	LPS (10 µg/mL) + IL-1β (50 ng/mL) + Vitamin A (7 µg/mL)

### qPCR

Isolation of total RNA from the cells was performed with a commercial kit (Thermo Fischer Scientific, K0732, GeneJet RNA Purification Kit, USA) following the manufacturer's protocol, and its amounts were determined with a UV-VIS spectrophotometry device (Biotek, NanoDrop Epoch, Microplate). cDNA was synthesized from the isolated RNA samples using a reverse transcription synthesis kit (Thermo Fischer Scientific, 4368814, High Capacity cDNA Reverse Transcription Kit, USA). qPCR analysis was performed using TaqMan probes (Thermo Fischer Scientific, 4448892, Gg07164124_m1, Chicken SAA, TaqMan Gene Expression Assays, USA) on the StepOne Plus device, repeating 40 times as two min at 50°C, 10 min at 95°C, 15 seconds at 95°C, one minute at 60°C. The change in mRNA expression level was calculated according to the 2^-ΔΔCt^ method using Ct values and GAPDH (445320, Gg03346982_m1, TaqMan Gene Expression Assays, USA) was used as the internal control gene. Changes in SAA gene expression were calculated semiquantitatively. Fold changes in expression levels were calculated based on the negative control group in which no substance was inoculated.

### ELISA

SAA protein concentration in the culture medium was measured with a commercial ELISA kit (Lifespan Biosciences, LS-F31849, Chicken SAA, USA). The analysis was carried out according to the manufacturer's instructions. Briefly, 100 μl of samples obtained from standard solutions and culture medium were added into the wells in triplicate. Then, the plates were covered with a protective film and incubated at 37°C for 90 min. At the end of the period, 100 μl of biotin-labeled antibody was added to the washed wells and incubated at 37°C for 60 min. 100 μl of HRP-streptavidin conjugate was added into the washed wells and the wells were incubated at 37°C for 30 min. At the end of the period, 90 μl of TMB substrate was added to the washed wells and incubated at 37°C for 30 min. Then, 50 μl of stop solution was added into the wells and the absorbance value of each well was measured at a wavelength of 450 nm. Using the trendline equation, normalization of the absorbance values read in all wells was performed and the amount of SAA corresponding to the absorbance values read in the standard wells was calculated.

### Statistical analysis

In the study, the Kolmogorov-Smirnov test was used to assess the normality of the data. The Kruskal-Wallis test was used for non-normally distributed parameters to compare SAA mRNA expression levels(2^-ΔΔCt^) data in CESF. Dunn's test was performed to identify the different groups and for multiple comparisons of the control group and other experimental groups. The Mann-Whitney U test was used for comparisons between two independent groups, and the Wilcoxon signed-rank test was applied for dependent groups (SAA mRNA expression at 24 and 48 h). SPSS for Windows (Release 20.0, Copyright SPSS Inc., Chicago, IL, USA) program was used to determine statistical values. SAA mRNA expression values are shown as the mean ± standard error. Statistical significance was defined as *p* < 0.001.

## Results

### Cell culture

**Fig 1:**
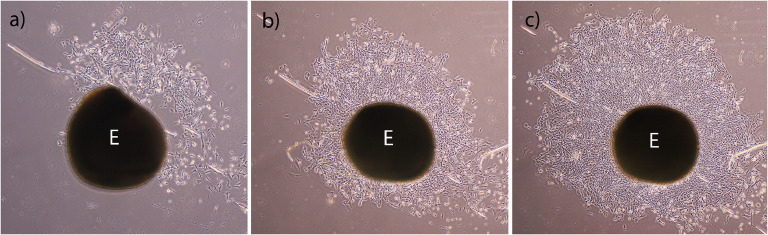
Cell growths from synovial tissue explant (E), inverted microscope. (a) Cell proliferation on the 3rd day. (b) Cell proliferation on the 4th day. (c) Cell proliferation on the 5th day.

The first cell proliferation started on the 3rd day and these cells were spindle-shaped. The proliferation continued rapidly in the following days and the cells completely filled the bottom of the plate wells (Fig. 1). The cells obtained from tissue explants by trypsinization were transferred to 25 cm^2^ flasks on the 7th day after cultivation. When transferred to 75 cm^2^ flasks in later passages, they were morphologically homogeneous and had a fibroblast-like cell appearance.

### Hemacolor staining, immunocytochemistry and carbon powder uptake test

**Fig 2:**
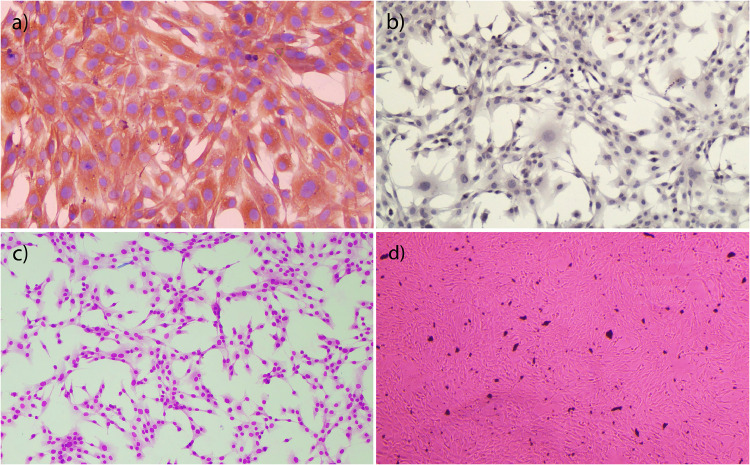
Characterization of cells. (a) Primary synovial fibroblasts, vimentin-positive immunostaining, DAB, x200. (b) Primary synovial fibroblasts, cytokeratin-negative immunostaining, DAB, x100. (c) Primary synovial fibroblasts, hemacolor staining, x100. (d) Carbon powder uptake test, non-phagocytosed carbon particles among primary synovial fibroblasts, inverted microscope.

While the cells obtained at the end of the 8th passage strongly expressed vimentin protein (Fig. 2a), no cytokeratin expression was observed (Fig. 2b). Hemacolor staining revealed many spindle-shaped, round-oval cells with basophilic colored nuclei and fibroblast-like morphology (Fig. 2c). In the carbon powder uptake test, it the isolated cells did not phagocytose carbon particles and the plates were not contaminated with macrophages (Fig. 2d). These results showed that the cells were synovial fibroblast cells (type B synoviocytes).

### qPCR

**Fig 3:**
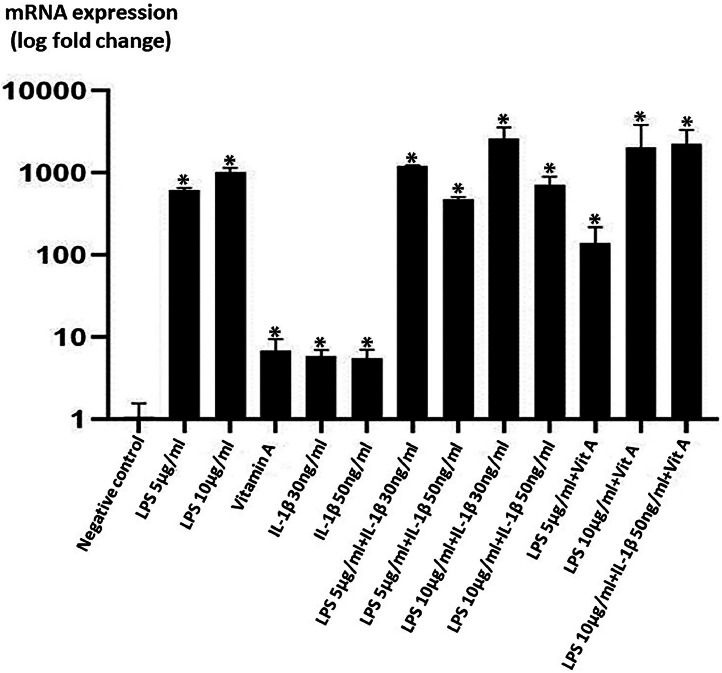
SAA mRNA expression at 24 h groups. *p < 0.001, (y axis shown logarithmically).

**Fig 4:**
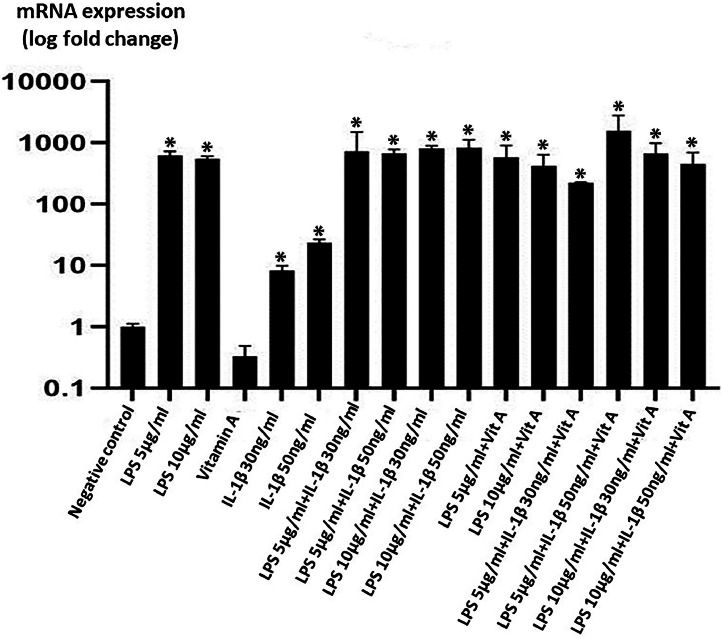
SAA mRNA expression at 48 h groups. *p < 0.001, (y axis shown logarithmically).

When the 24-hour groups were compared, the target gene expression values were significantly increased in the experimental groups compared to the negative control group (Fig. 3). The target gene expression values increased significantly in all 48-hour groups, except the vitamin A group, compared to the negative control group (Fig. 4). The 24th and 48th hour SAA mRNA expression values in CESF are given in Table 2.

**Table 2:** The 24th and 48th hour SAA mRNA expression values in CESF (*p < 0.001).

Groups	SAA mRNA expression at 24 h	SAA mRNA expression at 48 h
Negative control	1.1 ± 0.3	1 ± 0.1
LPS (5µg/ml)	612.2 ± 39*	625.4 ± 98.4*
LPS (10µg/ml)	1029.9 ± 112.6*	546.2 ± 56.5*
Vitamin A (7µg/ml)	6.8 ± 0.9*	0.3 ± 0.1
IL-1β (30ng/ml)	5.9 ± 1.1*	8.3 ± 1.1*
IL-1β (50ng/ml)	5.5 ± 1.5*	23.4 ± 2.8*
LPS (5µg/ml) + IL-1β (30ng/ml)	1212.4 ± 19.1*	734.6 ± 102.8*
LPS (5µg/ml) + IL-1β (50ng/ml)	476.6 ± 31.6*	680.3 ± 97.6*
LPS (10µg/ml) + IL-1β (30ng/ml)	2604.5 ± 476*	805.4 ± 89.2*
LPS (10µg/ml) + IL-1β (50ng/ml)	712.9 ± 184.1*	833.8 ± 94.7*
LPS (5µg/ml) + Vitamin A (7µg/ml)	139.4 ± 21.6*	582.6 ± 119.5*
LPS (10µg/ml) + Vitamin A (7µg/ml)	2040.7 ± 268*	419.4 ± 219.3*
LPS (5µg/ml) + IL-1β (30ng/ml) + Vitamin A (7µg/ml)	-	223.4 ± 4.3*
LPS (5µg/ml) + IL-1β (50ng/ml) + Vitamin A (7µg/ml)	-	1577.4 ± 326.4*
LPS (10µg/ml) + IL-1β (30ng/ml) + Vitamin A (7µg/ml)	-	672.7 ± 71.6*
LPS (10µg/ml) + IL-1β (50ng/ml) + Vitamin A (7µg/ml)	2246.8 ± 297.2*	453.6 ± 77.7*

### ELISA

No statistically significant increase in SAA levels was observed in the culture medium of CESF stimulated with LPS, IL-1β, and vitamin A.

## Discussion

Brown laying hens are used as models in experimental studies because they are prone to the development of amyloid arthropathy occurring, as a result of SAA accumulation in leg joints (Landman, 1999). Research on the intra-articular synthesis of SAA, which plays a role in amyloid arthropathy, continues (Blanco et al., 2016). *In vivo* studies on amyloid arthropathy in chickens revealed that especially the femora-tibial joint (Landman et al., 1997) and tibio-metatarsal joint (Landman and Feberwee, 2001; Sevimli et al., 2004, 2012) regions are affected. In this study, CESF isolated from the tibio-metatarsal joint region was used in the experiments. In studies in which chicken (Dayı et al., 2023; Liu et al., 2020; Upragarin et al., 2005), mouse (Zhao et al., 2016) and human (Fanqi et al., 2022; Xue et al., 1997) SF were isolated, a positive reaction to vimentin antibody was observed in immunocytochemical staining performed to confirm their mesenchymal origin, and in this study, vimentin expression was similar to the results in the literature. In addition, similar to other studies (Dayı et al., 2023; Upragarin et al., 2005), in this study carbon powder uptake test was applied to characterize the cells and the isolated cells were determined to be synovial fibroblasts.

It is a well-known fact that many factors play a role in the formation of amyloid arthropathy in chickens and accordingly SAA secretion increases (Landman et al., 1994; Ovelgonne et al., 2001; Sevimli et al., 2005). In studies conducted on the synthesis of SAA from several cells such as hepatocytes, macrophages and SF, it has been reported that SAA synthesis is induced by factors such as LPS (Gonçalves et al., 2012; Koziy et al., 2024; Teles et al., 2011; Upragarin et al., 2005), IL-1β and TNF-α (Jorgensen et al., 2000), Freund's adjuvant (Sevimli et al., 2012) and vitamin A (Abdelhamid et al., 2017; Sevimli et al., 2005). In addition, in some studies (Jacobsen et al., 2016; Koziy et al., 2024; Upragarin et al., 2005), SAA expression was observed in normal SF in negative control groups, and this was associated with the local synthesis of SAA extrahepatically from SF. Similarly, in our study, SAA expression was found in the negative control group.

LPS, obtained from the cell wall of gram-negative bacteria, is considered a strong bioactivator of the immune system and is preferred as an important inducer in the release of acute phase proteins and proinflammatory cytokines in inflammatory reactions (Li et al., 2015). Upragarin et al. (2005) found that in an approximately 35-fold increase was observed in SAA mRNA expression by SF after 6 h of stimulation with a dose of 5 μg/mL LPS, and a 40-fold increase was observed when a dose of 10 μg/mL LPS was applied. Zerega et al. (2004) reported that there was an approximately 7-fold increase in SAA mRNA expression in chicken embryonic chondrocytes 16 h after 10 μg/mL LPS induction. In an *in vivo* study in fish, with 10 μg/mL LPS application, SAA expression started to increase within 6 h, reached its maximum level at the end of 24 h, and decreased within 48 h (Gonçalves et al., 2012). When the results in the relevant literature are compared with the data obtained in our study, it is seen that acute phase response (APR) induced by different doses of LPS begins to take shape within 6 h, reaches its highest level within 24 h, and partially decreases within 48 h depending on the dose.

It is known that proinflammatory cytokines synthesized from activated cells at the site of inflammation mediate APR (Jensen and Whitehead, 1998). It has been noted that IL-1β, one of these cytokines, has important roles in arthritis pathogenesis through the regulation of inflammatory reactions, in addition to having direct catabolic effects on cartilage and SF (Goldring, 2000). Jacobsen et al. (2016) reported that proinflammatory cytokines, especially IL-1β, were the most effective inducer of SAA synthesis in SF and chondrocytes in horses. In the same study, an approximately 30-fold increase in SAA mRNA expression was observed in horse SF induced with 50 ng/mL IL-1β for 12 h, while a 130-fold increase was observed at the end of 24 h and a 20-fold increase at the end of 48 h. Sevimli et al. (2008) observed that IL-1β was significantly more effective than TNF-α in the induction of SAA. Ramadori et al. (1985) noted that as a result of IL-1β application to mouse hepatocytes, there was an increase in SAA level within 2 h, reaching its maximum level within 4 h and decreasing within 12 h. Jorgensen et al. (2000), in a study conducted on hepatocyte cells obtained from fish, applied 25 ng/mL IL-1β for 48 h and found that the level of SAA mRNA increased. In our study, the proinflammatory cytokine IL-1β, which is thought to be the most effective when used alone on SAA synthesis, was preferred and, unlike the results stated in the literature, it was determined that stimulation within 48 h caused a higher induction in CESF than stimulation within 24 h. It is thought that this observed difference may be due to the effect of IL-1β on SAA synthesis in chickens, the applied dose, duration, and species differences between animals.

There are studies on the roles of vitamin A, retinol and its active metabolite retinoic acid in the formation of amyloidosis. De Buck et al. (2016), in a study they conducted in mice, suggested that vitamin A is necessary for the formation of SAA gene expression. Derebe et al. (2014) showed that SAA transcription decreased in mice fed a diet deficient in vitamin A and noted that SAA is a retinol-binding protein that mediates the transport of retinol during infections. It is suggested that vitamin A, which is thought to have an effect on immunity, including the functions of leukocytes and the expression of cytokines, is also effective in amyloid formation (Dillehay et al., 1988; Göttgens and Green, 1995; Turpin et al., 1990). Sevimli et al. (2005) observed the most severe SAA release in the vitamin A group in chickens in which amyloid arthropathy was induced experimentally with Freund's adjuvant. They also found that there was a negative correlation between serum retinol and SAA levels and amyloid accumulation in the tissues, and they suggested that vitamin A taken with nutrition passes into the tissues and mixes with the amyloid structure, therefore the serum retinol level was low (Sevimli et al., 2005). In our study, while an increase in SAA expression was observed in the group administered with vitamin A for 24 h, a decrease of approximately 75 % was observed in the group administered for 48 h compared to the negative control group. According to these findings, it is thought that the CESF isolated in the study needed vitamin A for their nutrition and development in the culture medium, and as a result, they might have used vitamin A applied to the culture medium.

It has been suggested that the signaling network of cytokines, which play an important role in the pathogenesis of joint diseases, is very complex and therefore examining it alone cannot fully reflect the interactions of all stimuli involved in the disease process (Jacobsen et al., 2016). Additionally, it has been noted that proinflammatory cytokines are synthesized from SF in joint diseases and contribute greatly to the initiation and maintenance of inflammation (Bartok and Firestein, 2010; Sevimli et al., 2008). Jorgensen et al. (2000) found that in addition to SAA synthesis in fish cells, which was induced separately by LPS and IL-1β, there was also IL-1β synthesis. Similarly, He et al. (2020), in an *in vivo* study conducted in mice, found an increase in the expression of proinflammatory cytokines as a result of LPS induction. This shows that proinflammatory cytokines are also released into the environment as a result of triggering during APR. Landman et al. (1998), demonstrated that IL-1β is an important inducer of SAA in chickens. SFs, which play an important role especially in joint diseases, cause an ongoing inflammatory reaction by releasing proinflammatory cytokines during APR (Yoshitomi, 2019), and it is suggested that this situation plays an active role in SAA synthesis, especially locally (Liu et al., 2021; Upragarin et al., 2005). Therefore, it is thought that the reason for the highest SAA mRNA expression 24 h after LPS + IL-1β application in our study is that IL-1β acts in combination with an important SAA inducer such as LPS.

Landman et al. (1998), showed that IL-1β is an important inducer of SAA. SFs, which play an important role especially in joint diseases, cause an ongoing inflammatory reaction by releasing proinflammatory cytokines during APR (Ritchlin, 2000; Yoshitomi, 2019), and it is suggested that this situation plays an active role in SAA synthesis, especially locally (Blanco et al., 2016; Liu et al., 2021; Upragarin et al., 2005). Therefore, it is thought that the reason for the highest SAA mRNA expression 24 h after LPS + IL-1β application in our study is that IL-1β acts in combination with an important SAA inducer such as LPS.

Sevimli et al. (2004), in their experimentally induced amyloid arthropathy model in chickens, observed that the most severe SAA release was in the group administered with vitamin A together with *E. faecalis*. Abdelhamid et al. (2017) examined the release of SAA induced by LPS and LPS + retinoic acid to horse peripheral mononuclear blood cells. They found that SAA synthesis from the cells was less in the LPS + retinoic acid-induced group than in the LPS-only group. They explained this situation by the fact that retinoic acid has an immunomodulatory effect on cells (Abdelhamid et al., 2017). In our study, the highest increase among the groups administered with LPS + vitamin A was detected in the group administered 10 μg/mL LPS and vitamin A for 24 h. According to these results, it was concluded that APR was at its maximum level within 24 h of application and decreased within 48 h.

He et al. (2020), found that proinflammatory cytokine expression decreased after vitamin A treatment in mice. It has been reported that LPS, which has various effects on the host, induces cells to produce pro-inflammatory cytokines with its harmful stimulus effect, initiating APR (Kent et al., 1998). However, vitamin A reduces cytokine synthesis, causing the loss of proinflammatory effects in cells and the formation of an immunomodulatory effect (Abdelhamid et al., 2017). In this study, the fact that SAA expression was observed to be lower in the combined groups compared to the LPS or LPS + IL-1β applied groups, as a result of vitamin A producing the same effects and reducing IL-1β synthesis, can be explained by the results stated in the literature. According to the findings in this study, we think that SAA expression in CESF depends especially on the concentration of LPS, and that IL-1β and vitamin A play an important role in the induction of APR in the early period, especially depending on the dose of LPS, as stated in the literature on SAA expression alone (Abdelhamid et al., 2017; Kent et al., 1998).

It is known that ELISA measured SAA values in studies (Sevimli et al., 2008, 2013) conducted under *in vitro* conditions. In addition, Upragarin et al. (2005), measured the SAA protein concentration in the culture medium of SF stimulated with different concentrations of LPS in laying hens under *in vivo* conditions by ELISA, and reported that an increase of 100 ng/mL in SAA levels occurred at the end of 12 h, 200 ng/mL at the end of 24 h, and 400 ng/mL at the end of 48 h. However, in our study, no significant change was observed in the SAA protein level in the culture medium of the experimental groups with ELISA. This suggests that CESF produces SAA protein in the culture medium at a level that cannot be detected by the ELISA, or may be due to the fact that the isolated cells used in the study were of the embryonic period.

The current findings obtained in this study show that the IL-1β and vitamin A have a positive effect on SAA production in LPS-stimulated CESF. CESF obtained from brown laying hen embryos, which are susceptible to amyloid arthropathy, represent a suitable study model for AA amyloidosis. It was concluded that using SF isolated from leg joints in embryos as an *in vitro* culture model can make significant contributions to the investigation of the pathogenesis of leg diseases that cause significant health problems and economic losses in the poultry industry. In addition, the results supported studies suggesting that SF are key cells directing pathological processes and reporting that the increase in SAA mRNA expression, which is thought to be a reliable parameter in monitoring bird health, has an important role in the formation of amyloid arthropathy. In future studies on amyloid arthropathy in chickens, it is recommended that SAA levels be measured more frequently with molecular methods in order to fully determine the role of SAA concentration in pathogenesis. In addition, it was concluded that it would be appropriate to examine SAA synthesis in the acute and chronic periods in more detail, depending on the agents and dosage applied in future studies.

## Declaration of competing interest

The authors declare that they have no known competing financial interests or personal relationships that could have appeared to influence the work reported in this paper.
